# Retinoic Acid Mediates Long-Paced Oscillations in Retinoid Receptor Activity: Evidence for a Potential Role for RIP140

**DOI:** 10.1371/journal.pone.0007639

**Published:** 2009-10-28

**Authors:** Kelly C. Heim, Joshua J. Gamsby, Mary P. Hever, Sarah J. Freemantle, Jennifer J. Loros, Jay C. Dunlap, Michael J. Spinella

**Affiliations:** 1 Department of Pharmacology and Toxicology, Hanover, New Hampshire, United States of America; 2 Department of Genetics, Dartmouth Medical School, Hanover, New Hampshire, United States of America; 3 Norris Cotton Cancer Center, Dartmouth Hitchcock Medical Center, Hanover, New Hampshire, United States of America; Istituto Dermopatico dell'Immacolata, Italy

## Abstract

**Background:**

Mechanisms that underlie oscillatory transcriptional activity of nuclear receptors (NRs) are incompletely understood. Evidence exists for rapid, cyclic recruitment of coregulatory complexes upon activation of nuclear receptors. RIP140 is a NR coregulator that represses the transactivation of agonist-bound nuclear receptors. Previously, we showed that RIP140 is inducible by all-*trans* retinoic acid (RA) and mediates limiting, negative-feedback regulation of retinoid signaling.

**Methodology and Findings:**

Here we report that in the continued presence of RA, long-paced oscillations of retinoic acid receptor (RAR) activity occur with a period ranging from 24 to 35 hours. Endogenous expression of RIP140 and other RA-target genes also oscillate in the presence of RA. Cyclic retinoid receptor transactivation is ablated by constitutive overexpression of RIP140. Further, depletion of RIP140 disrupts cyclic expression of the RA target gene HOXA5. Evidence is provided that RIP140 may limit RAR signaling in a selective, non-redundant manner in contrast to the classic NR coregulators NCoR1 and SRC1 that are not RA-inducible, do not cycle, and may be partially redundant in limiting RAR activity. Finally, evidence is provided that RIP140 can repress and be induced by other nuclear receptors in a manner that suggests potential participation in other NR oscillations.

**Conclusions and Significance:**

We provide evidence for novel, long-paced oscillatory retinoid receptor activity and hypothesize that this may be paced in part, by RIP140. Oscillatory NR activity may be involved in mediating hormone actions of physiological and pathological importance.

## Introduction

The retinoids are derivatives of vitamin A that influence development, differentiation and homeostasis and are pharmacologically valuable in the prevention and treatment of select neoplasms [1]–[3]. The biological effects of retinoids are mediated by retinoid receptors, members of the nuclear receptor (NR) superfamily of ligand-dependent transcription factors [4]. The functional receptor unit for all-*trans* retinoic acid (RA) is the heterodimer RAR/RXR, which binds consensus retinoic acid response elements (RAREs) in the proximal promoters of RA target genes [4].

Positive and negative regulation of gene expression by NRs is contingent upon coordinated interaction with coregulatory molecules (coactivators and corepressors), which nucleate precisely organized chromatin remodeling complexes [5]. In the absence of ligand, interaction of the ligand-binding domain with corepressors such as NCoR1 or NCoR2/SMRT mediates transcriptional repression via recruitment of complexes with enzymatic histone modifying activity [5]. Upon ligand binding, this complex dissociates and the binding of coactivators such as SRC1/NCoA1, SRC2/TIF2/NCoA2 or SRC3/AIB1/NCoA3 enable a transcriptionally permissive chromatin configuration through histone acetylation and other covalent modifications [5].

RIP140 is distinct from classic corepressors due to its preferential interaction with ligand-bound NRs and is therefore regarded as the founding member of a distinct class of coregulators that includes LCoR and PRAME [6], [7]. RIP140 directly recruits histone deacetylases and C-terminal binding protein, a transcriptional repressor, to ligand-bound NRs to repress transcription of RA target genes [8], [9].

Our prior work demonstrated that RIP140 itself is a transcriptional target of RA and that it functionally limits retinoid signaling, comprising a negative feedback mechanism that is relevant to the biological activity of RA [10], [11]. Depletion of RIP140 through small interfering RNA (siRNA) sensitizes human embryonal carcinoma cells to the antiproliferative and differentiating effects of RA [11], [12]. We have also demonstrated on a genome-wide scale that RIP140 discriminates between different classes of RA target genes [12].

It is recognized that NR signaling is temporally organized at multiple levels [5], [13]. Posttranslational modifications and proteolysis of NR complex components has been shown to result in rapid cycles of NR-dependent coregulator recruitment and transcription in the constant presence of ligand [5], [13]. In this report we provide evidence for the existence of additional, long-paced oscillations in RAR activity. The data supports the existence of a previously unrecognized form of RAR signaling dynamics that may extend to other NRs and participate in the homeostasis of hormone action.

## Materials and Methods

### Cell culture, siRNA and transfection protocols

NT2/D1, MCF-7, T47D, U2OS and NIH-3T3 cells were purchased from American Type Culture Collection (Manassas, VA). COS-1 cells were a gift from Dr. John Hwa (Dartmouth Medical School). MCF-7 and T47D cells were cultured in DMEM/F12 (50:50) and all other cell lines were cultured in DMEM (Gibco).

For estrogen treatments, MCF-7 cells were cultured in phenol-free medium beginning 24 hours before drug treatment. 17-β−estradiol (E2) and RA were purchased from Sigma. 1,25-dihydroxyvitamin D_3_ was purchased from Biomol. Dexamethasone and rosiglitazone were gifts from Dr. Joshua Hamilton (Dartmouth Medical School) and Dr. Michael Sporn (Dartmouth Medical School), respectively. RA was stored protected from light in liquid nitrogen as a 10 mM stock solution in dimethyl sulfoxide (DMSO). 4-[(E)-2-(5,6,7,8-Tetrahydro-5,5,8,8-tetramethyl-2-naphthalenyl)-1-propenyl] benzoic acid (TTNPB, Tocris Bioscience) and bexarotene (LC Laboratories) were stored away from light at −20°C as 10 mM stock solutions in DMSO. All other drugs were dissolved in ethanol (EtOH) and stored away from light at −20°C. For the extended time courses of RA treatment, 1×10^6^ NT2/D1 cells were plated in 10 cm plates and maintained in charcoal-absorbed sera containing media for 48 hours to deplete endogenous NR ligands. After 48 hours, media was replaced with serum-free media containing 2.5 µM α-amanitin (Sigma) for 2 hours. Cells were washed and DMEM containing 10% charcoal-absorbed sera and 1 µM RA was added. RA remained on the cells until harvest.

For siRNA experiments, custom designed siRNA duplexes for RIP140, NCoR1 and SRC1 were purchased (Dharmacon, Lafayette, CO). Human RIP140 siRNA is of sequence 5′-CAAACAGGAUAGCACAUUA-3′ and corresponds to the RIP140 cDNA beginning 1986 bp downstream of the ATG start codon. Human NCoR1 siRNA is of sequence5′-AAAGUCGUUAUCCUCCUCACU-3′ and corresponds to the NCoR1 cDNA beginning 49 bp downstream of the ATG start codon. Human SRC1 siRNA is of sequence 5′-AGGAGACAGGUUACUUCUG-3′ and corresponds to the cDNA beginning 1467 bp downstream of the start codon. The control was the Scrambled siRNA of sequence 5′-GCGCGCUUUGUAGGAUUCG-3′ from Dharmacon. For transient siRNA experiments, log-phase cells were transfected with 150 nM siRNA using OligofectAMINE (Invitrogen) as described [11], [12]. Briefly, 2−3×10^6^ cells per 10 cm plate were transfected with 150 nM siRNA for 16 h. Cells were split the following day onto 10 cm plates with charcoal-absorbed sera media containing either 1 µM RA or DMSO. Cells remained in this media until the time of harvest.

### Conventional reporter assays

The pSG5-RIP140 expression plasmid has been described previously [10], [11]. The pCMX-PPARγ expression plasmid and the reporter constructs PPRE-tk-Luc and VDRE-tk-Luc were kindly provided by Dr. Michael Sporn (Dartmouth Medical School). The Ebo-RXR expression plasmid was a gift from Dr. Ethan Dmitrovsky (Dartmouth Medical School). The pCI-mGR expression plasmid contains the full-length murine glucocorticoid receptor sequence and was kindly provided by Dr. Lynn Sheldon (Dartmouth Medical School). For reporter assays, COS-1, NT2/D1, or T47D human breast cancer cells were plated at 0.2×10^6^ cells per well of a 6-well plate and transfected with 2 µg total DNA using Polyfect™ (Qiagen) according to the manufacturer's instructions. Transfections were performed in triplicate for each condition.

For the PPRE-Luc experiment, COS-1 cells were transfected with 0.1 µg PPRE-Luc, 0.125 µg CMV-PPARγ, 0.125 µg Ebo-RXRA, 0.15 µg pSG5-RIP140, and 0.1 µg Renilla luciferase plasmid (pRL-tk) per well. For the GRE-luc experiment, COS-1 cells were transfected with 0.1 µg GRE-Luc, 0.1 µg GR, 0.1 µg pRL-tk, and 0.45 µg pSG5-RIP140. The dual-luciferase assay system (Promega) was used to normalize firefly luciferase activity against renilla luciferase activity. In all conventional reporter transfections, total DNA was brought to 2 µg per well with salmon sperm DNA. Media containing the transfection mixture was removed after 16 hours and charcoal-absorbed sera media containing 1 µM rosiglitazone, 1 µM dexamethasone, 1 µM vitamin D_3_ or vehicle control (DMSO or ethanol) was added. Cells were lysed 24 hours following drug treatment and luciferase activity was determined. Data points represent the average of triplicate transfections.

### Real-time reporter assays

The RARE-tk-Luc reporter consists of a fusion of two RARE sequences to a minimal herpes simplex virus-thymidine kinase promoter and luciferase and has been used previously [10], [11]. For transient transfection real-time reporter assays, 1.5×10^4^ NT2/D1 cells were plated in triplicate 35 mm plates in charcoal absorbed sera media 24 hours prior to transfection. Cells were transfected with 1.75 µg total DNA per plate consisting of 0.75 µg RARE-tk-Luc and 1 µg insertless pSG5 or pSG5-RIP140. For the siRNA condition, 150 nM RIP140 siRNA was transfected with 0.75 µg RARE-tk-Luc with otherwise identical transfection parameters as the other three conditions. Transfections were performed using Polyfect (Qiagen) according to the manufacturer's directions. After a 16 hour incubation with the transfection mixture, cells were washed with PBS and 2 ml of Leibovitz's L15 media (Gibco) containing 10% charcoal-absorbed FBS, 1% penicillin/streptomycin, 10 mM HEPES, 0.1 µM luciferin and 1 µM RA or DMSO was added to each plate. Plates were sealed with silicone grease and glass cover slips and immediately transferred to a LumiCycle (Actimetrics, Wilmette, IL) for data collection as previously described [14].

For real-time reporter assays employing U2OS and NIH-3T3 cells containing a stably integrated RARE-tk-luciferase cassette, cells were plated to confluency in triplicate 35 mm plates in charcoal absorbed sera media. The following day, cells were transferred to 0.5% charcoal absorbed sera media for 2 days. For one arm of the U2OS experiment cells were pretreated with 2.5 µM α-amanitin in serum-free media for 2 hours. All cells were then washed with PBS and 2 ml of Leibovitz's L15 media containing 10% charcoal-absorbed FBS, 1% penicillin/streptomycin, 10 mM HEPES, 0.1 µM luciferin and 10 µM RA or 5 µM TTNPB plus 5 µM bexarotene was added to each plate. Plates were sealed and transferred to the LumiCycle for data collection. The data collection interval was set for 6 collections/hour. The LumiCycle is housed in a standard tissue culture incubator at 36°C instead of 37°C to minimize background counts. The data was collected up to 72 to 100 hours and analyzed with the LumiCycle Analysis software (Actimetrics). For the U2OS and NIH-3T3 cells, the data were baseline subtracted based on a running average as previously described [14].

The stable RARE-tk-Luc U2OS and NIH3T3 cells were generated using a lentivirus expressing luciferase under control of the above described RARE-tk promoter and enhancer. The lentiviral RARE-tk-Luc plasmid was obtained by cloning the RARE-tk portion of the RARE-tk-Luc plasmid in place of the β-catenin response element in the Topflash lentiviral vector obtained from Dr. Karl Willert (University of California). DNA sequencing confirmed successful cloning.

### Stable shRNA

For generation of stable RIP140 knockdown in NT2/D1 cells, a RIP140 shRNA lentiviral construct with a sense strand sequence of 5′-GCGGAGAAGAATGAGTATGAA-3′ was purchased (Open Biosystems, clone ID TRCN0000019779). A pLKO.1-shRNA lentiviral construct targeting eGFP of sense strand sequence 5′-TACAACAGCCACAACGTCTAT-3′ was also purchased (pLKO.1-eGFP, Sigma) and used as a control. Lentiviral stocks were generated from 293T cells and psPAX2 and pMD2G packaging and envelop vectors using standard protocols. Viral stocks were added to NT2/D1 cells plated at 0.2×10^6^ cells/well per 6 well plates. The lentiviral stock remained on the cells for 24 h. Cells were split onto 10 cm plates in selection media containing 0.5 µg/ml puromycin. Selection continued for 48 hours, after which no viable cells remained in a mock-transduced control plate. The puromycin-resistant cell pools were used for experiments.

### Northern hybridization, semiquantitative RT-PCR and realtime RT-PCR

Total RNA was isolated using TriReagent (Molecular Research Center). Northern hybridizations were performed with 5 µg RNA as described previously [10], [11] using radiolabeled 500–1100 bp DNA fragments from RIP140, SRC1, NCoR1 or RARB. The RIP140 probe was a 1000 bp fragment from a BglII/XhoI digest of the pSG5-RIP140 plasmid [10], [11]. The SRC1 probe was a 1071 bp fragment from a BamHI/HindIII digest of the pCR3.1-hSRC1A construct kindly provided by Dr. Bert O' Malley (Baylor College of Medicine). The NCoR1 probe was a 1075 bp fragment of the pCMX-NCoR construct provided by Dr. Ronald Evans (The Salk Institute). For semiquantitative PCR, cDNA was synthesized from 5 µg RNA using Superscript II reverse transcriptase (Invitrogen), and CYP24A1 or RIP140 expression was analyzed by PCR using Taq polymerase (Invitrogen) to cycle numbers within the linear range as described [12]. For realtime RT-PCR, cDNA was synthesized from 2 µg RNA using TaqMan reverse transcription reagents (Applied Biosystems) and RIP140, RARA, RARB, SRC1, NCoR1, and HOXA5 were amplified from 25 ng cDNA per reaction using SYBR Green reagent (Applied Biosystems). RIP140 transcripts containing exon 1a and exon 1b were amplified using TaqMan primers and reagents (Applied Biosystems). Gene expression data was normalized to GAPDH. Primer sequences are listed in Figure S1. Data was collected and analyzed with 7500 Fast System Sequence Detection Software version 1.4 (Applied Biosystems).

### In silico analysis of the RIP140 locus

The Evolutionarily Conserved Regions Browser (http://ecrbrowser.dcode.org) is a graphical interface described by Loots *et al*. [15]. This resource was used to search the RIP140 gene for consensus transcription factor response elements that exhibit a high level of evolutionary conservation. A 550 bp region of the RIP140 ECR was amplified by genomic PCR and cloned into a TK-luciferase construct. PCR primers were 5′ TAGGTAAACCCTCTCTCCAGAAT and 5′ ATCCTCTCCATCAACTACAAAGC. DNA sequencing confirmed that the correct sequence was cloned. The cloned sequence can be found under the previously existing NCBI accession number, NT_011512.

## Results

### RAR transactivation is cyclic and sensitive to RIP140 depletion and overexpression

**Figure 1:**
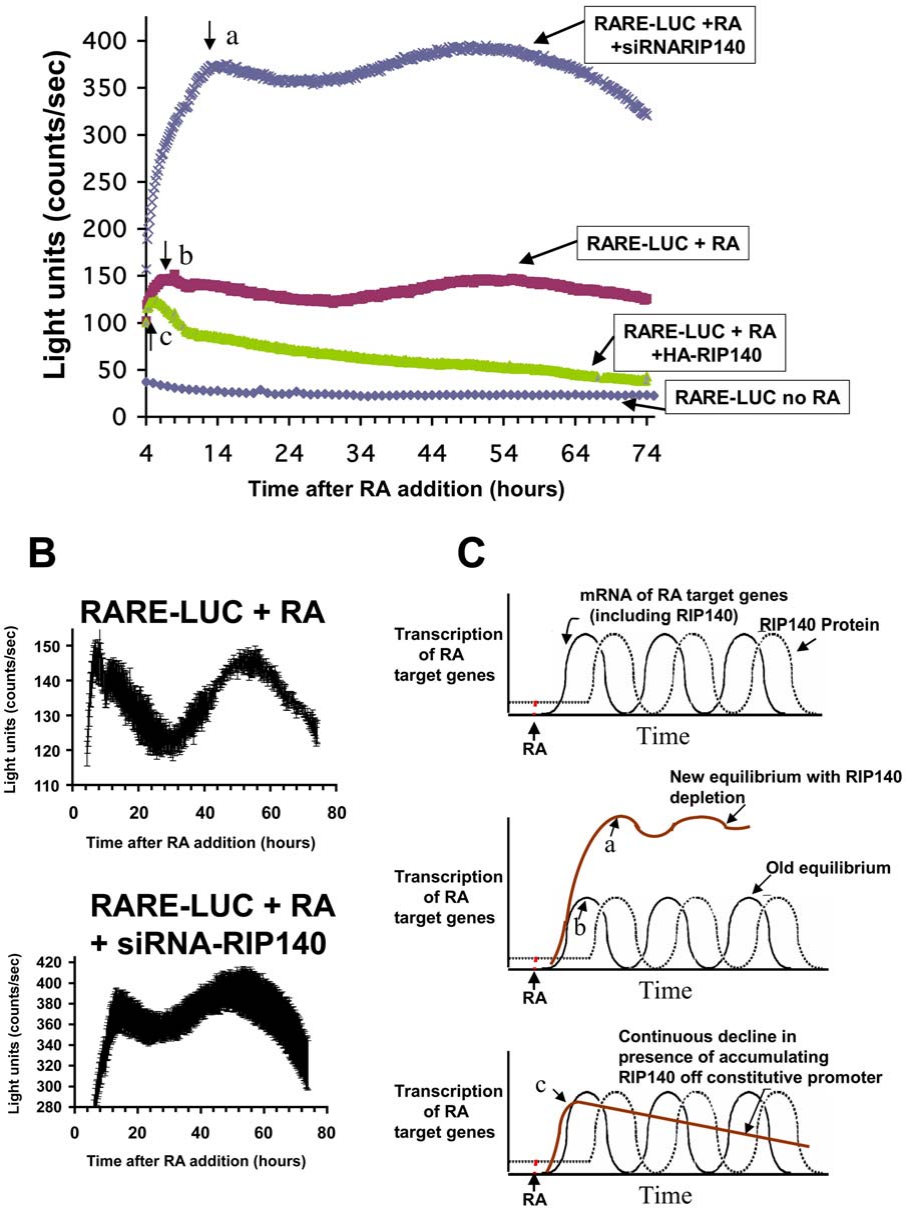
RAR-dependent transactivation is cyclic in the continued presence of RA and is sensitive to RIP140 depletion and overexpression. A, Actively proliferating NT2/D1 cells were transiently tranfected with an RARE-tk-luciferase reporter and either a control insertless expression plasmid (RAR-LUC + RA, RARE-LUC no RA), a RIP140 expression plasmid driven by the SV40 promoter-enhancer (RARE-LUC + RA + HA-RIP140) or RIP140 siRNA (RARE-LUC + RA + siRNA-RIP140). Cells were then treated with RA (1 µM) or vehicle and luciferase activity was monitored in live cells every 10 minutes for 74 hours using a LumiCyle luminometer. Each date line represents the average of three biological replicate plates. The experiment was performed twice with similar results. B, Two of the data lines in (A), RARE-LUC + RA and RARE-LUC + RA + siRNA-RIP140 are depicted with zoomed scaling of y-axis. Data points are represented by the error bars (S.E.M.) of the replicate plates. C, Hypothetical model representing one interpretation of results in (A), see text for details.

We sought to characterize RAR activity over an extended time course in the constant presence of RA. To this end, NT2/D1 cells were transiently transfected with a RA-responsive luciferase reporter (RARE-tk-Luc), and RA-mediated RARE transactivation was monitored in real time with a LumiCycle instrument. RA-dependent reporter activity was monitored in biological triplicate every 10 minutes for 76 hours following addition of RA. RA treatment resulted in oscillatory RAR activity with an extended period (Figure 1A and 1B). We believe this is the first demonstration of long-timed oscillations in RAR activity in the continued presence of RA.

Our previous studies using conventional reporter assays established that RIP140 siRNA enhances RAR activation of endogenous RA target genes and heterologous RARE-containing promoters while RIP140 overexpression represses this activation [10]–[12]. These studies involved only one time point (generally 24 or 48 hours). We sought to characterize whether the oscillations in RAR activity were influenced by RIP140 by employing RIP140 siRNA or a RIP140 expression plasmid (HA-RIP140). RARE–dependent oscillations in the constant presence of RA were completely abolished by RIP140 overexpression driven by a constitutive SV40 promoter that is not subject to RA regulation (Figure 1A). The linear decline in RAR activity may reflect accumulation of RIP140 over the course of the experiment.

RIP140 siRNA produced an overall amplification of RARE-luciferase activity as compared to RA treatment alone. RAR activity was robustly enhanced by RIP140 siRNA at all time points beginning at 4 hours (Figure 1A). However, the oscillations were slightly perturbed with a suggestion of a partially “flattened” response (Figure 1B). Our previous work indicated that RIP140 siRNA does not completely ablate basal or RA-induced RIP140 expression in NT2/D1 cells [11], [12]. This residual RIP140 expression may account for the retention of RAR oscillation with siRNA treatment.

Based on these data the following hypothetical model depicted in Figure 1C is proposed. We have previously found that RA induction of RIP140 constitutes a negative feedback mechanism that limits and fine-tunes retinoid signaling [10]–[12]. The negative feedback predicts that upon RA addition, RIP140 should accumulate until a level sufficient to inhibit RAR activity is attained. RIP140 expression should then decline, allowing a recovery of RAR transactivation activity and the re-expression of RIP140.

A relationship between the time required to reach the initial peak of RARE activity and RIP140 status was also evident. With RIP140 overexpression the peak was reached most rapidly, at 5 hours (“c” in Figure 1A), whereas with endogenous or intermediate RIP140 levels, the peak was reached at 7 hours (“b” in Figure 1A). With RIP140 depletion, the peak was reached at 13 hours (“a” in Figure 1A).

### RAR activity oscillates in U2OS cells from a stably integrated promoter

To further examine RAR oscillatory activity, U2OS cells containing a stably integrated RARE-tk-luciferase cassette were utilized (Figure S2). In contrast to the NT2/D1 cells which have decreased viability upon prolonged confluence, U2OS cells were allowed to grow to confluence and serum-starved for 2 days prior to ligand addition. This allowed for the assessment of time-dependent nuclear receptor activity independent of the cell cycle. In addition to RA, U2OS cells were also treated with a combination of the retinoid analogs, TTNPB and bexarotene, which are RAR and RXR selective agonists, respectively. These ligands were chosen because they are reported to be more stable than RA and because RIP140 has been reported to more strongly bind to the RAR/RXR heterodimer when both RAR and RXR are engaged with ligand [16]. In addition, a group of cells were first treated with 2.5 µM α-amanitin for 2 hours prior to TTNPB and bexarotene treatment in an attempt to “transcriptionally synchronize” the cells [17]–[19]. For all treatment protocols, an initial peak in RARE-luciferase activity was evident after ∼10 hours of retinoid treatment followed by a second peak (35–45 hours) and a third peak (∼70 hours), resulting in a period ranging from 25–35 hours (Figure S2A). There was little difference between the RA and bexarotene/TTNPB treatments, and α-amanitin pretreatment did not affect the results. The amplitude of the oscillations dampened over the duration of retinoid treatment (Figure S2A). Dampening is often seen in biological oscillators and depends on the relative values of feedback parameters and also results from progressive asynchronization and averaging over a cell population [20]. No oscillations were seen when cells were treated with the vehicle control DMSO in place of RA (data not shown). These data indicate that RAR oscillatory activity from an integrated reporter can be maintained in the constant presence of RA even in cells that have left the cell cycle and are no longer proliferating.

### RAR activity oscillates in murine fibroblasts

We next examined the dynamics of RAR activation in a third cell context, NIH-3T3 cells stably integrated with RARE-tk-luciferase. NIH-3T3 cells were confluent and serum starved and therefore not proliferating during the course of bexarotene and TTNPB treatment. Luciferase activity first peaked between 8 and 12 hours, followed by two subsequent peaks at 26–32 and 60–65 hours for a period of ranging from 25–30 hours (Figure S2B). As seen above in U2OS cells, the amplitude of the oscillations dampened over the duration of the retinoid treatment.

### RIP140 oscillates in the constant presence of RA

**Figure 2:**
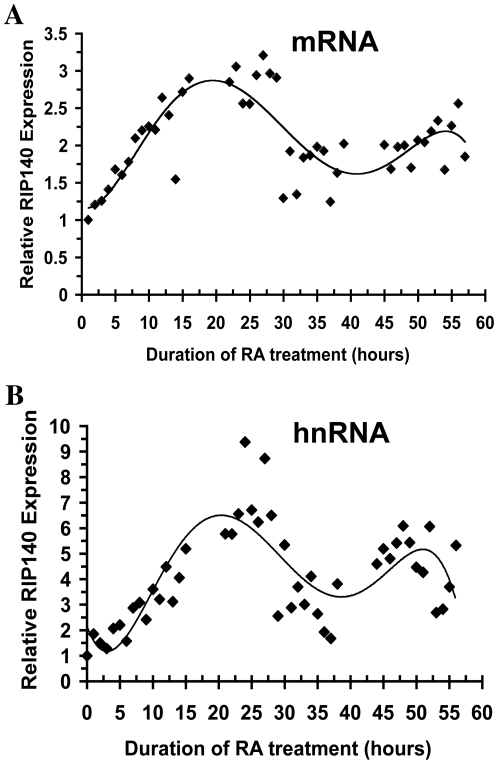
Endogenous RIP140 transcripts oscillate in the continued presence of RA. NT2/D1 cells were pretreated with alpha-amanitin (2.5 µM) for 2 hours and then treated with RA (1 µM) for the indicated time points. RIP140 expression was monitored by quantitative real-time PCR using primers in A, the processed transcript or B, in an intron of the unspliced heteronuclear transcript. Data was normalized to GAPDH. Trend line is the 5^th^ order polynomial. One experiment of three with similar results is presented.

The negative feedback predicts that RIP140 should cycle in the constant presence of RA. To investigate whether RIP140 mRNA cycles with continual RA exposure, we pretreated NT2/D1 cells with the RNA polymerase II inhibitor, alpha-amanatin, for 2 hours and then treated the cells with RA over an extensive 57-hour time course. Treatment with alpha-amanatin is a common strategy to synchronize NR activity and has been used extensively to measure rapid ligand-dependent coactivator complex recruitment to promoters of NR target genes [17]–[19]. Steady-state RIP140 mRNA increased over the first 25 hours of RA treatment, followed by a steady decline over the next 10 hours and a partial recovery from 40 to 57 hours of RA treatment (Figure 2A). Primary unspliced transcript levels also cycled with increased amplitude (Figure 2B). The oscillations were not observed in cells treated with alpha-amanatin alone. These oscillations are consistent with the negative feedback model and long 24–30 hour periodicity of RIP140 expression is consistent with long-timed transcriptional-translational feedback. Further it is possible that the oscillation is circadian in nature since the period partially overlaps with a circadian period and given the known relationship between NRs and the circadian clock apparatus [21]. Whether RIP140 would eventually decline over time due to negative feedback of RAR activity would require longer time points.

### Endogenous RA target genes oscillate in the constant presence of RA

**Figure 3:**
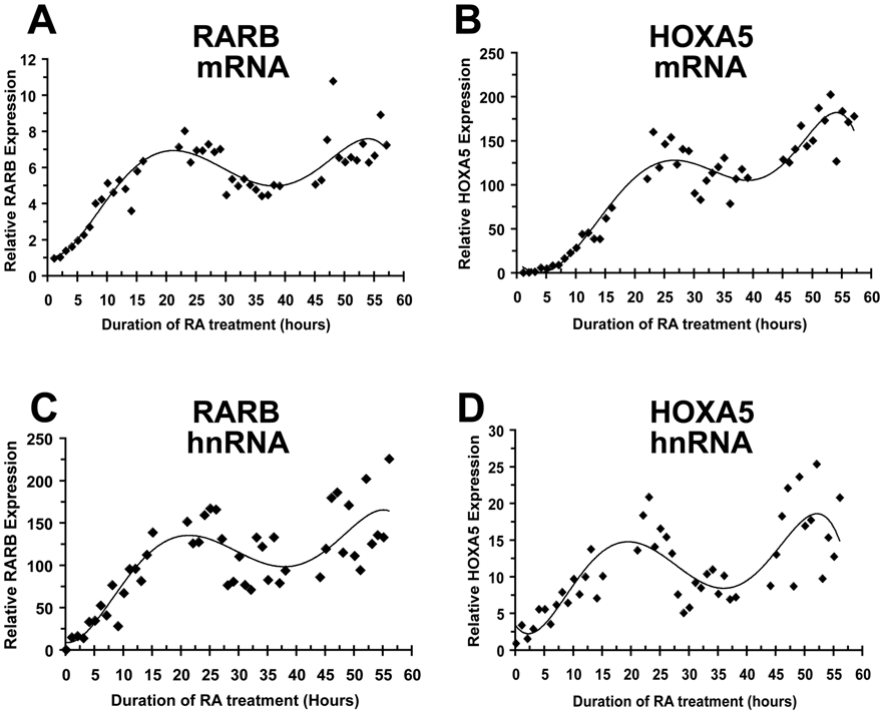
Endogenous expression of RA target genes oscillate in the continued presence of RA. Actively proliferating NT2/D1 cells were pretreated with alpha-amanitin (2.5 µM) for 2 hours and then treated with RA (1 µM) for the indicated time points. RARB (A, C) and HOXA5 (B, D) expression was monitored by quantitative real-time PCR using primers in the processed transcript (A, B) or in an intron on the unspliced heteronuclear transcript (C, D). Data was normalized to GAPDH. Trend line is the 5^th^ order polynomial except for B, 6^th^ polynomial. The experiment was performed three times with similar results.

Cyclic RAR activation suggests that endogenous RA target genes should also oscillate with continued RA exposure. As depicted in Figure 3, both primary and steady state RARB transcripts oscillate with a period of approximately 25–30 hours in the continued presence of RA. HOXA5 expression was also cyclic with a similar period as RARB (Figure 3). For both RARB and HOXA5, oscillations of primary transcript levels, which are more indicative of transcription rate, were more pronounced as compared to steady state mRNA levels.

### RIP140 depletion alters cyclic RA target gene expression

**Figure 4:**
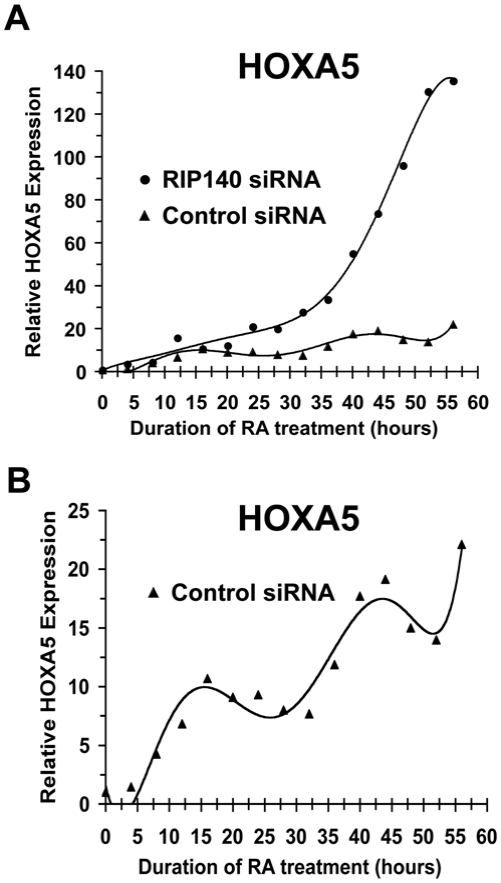
RIP140 depletion alters the cyclic pattern of HOXA5 expression. A, NT2/D1 cells were treated with RIP140 siRNA or control siRNA and exposed to RA (1 µM) for the indicated time points. HOXA5 expression was measure by quantitative real-time PCR specific for unspliced heteronuclear transcripts. B, Data in (A) for control siRNA treated cells with zoomed scaling of y-axis. Data was normalized to GAPDH. Trend line is the 6^th^ order polynomial.

RIP140 constitutes a negative feedback mechanism to limit RA signaling and is expressed in a cyclic fashion with continued RA treatment. We asked whether RIP140 depletion perturbs the oscillation of an RA target gene. The effect of RIP140 siRNA on the cyclic expression of HOXA5 was explored. NT2/D1 cells were transiently transfected with control or RIP140 siRNA. Cells were subjected to a 56-hour time course of RA treatment. RNA was harvested every 4 hours and gene expression was determined by real-time PCR. Under these conditions RA induced a clear oscillation in HOXA5 expression with a period approximating 30 hours in control siRNA treated cells (Figure 4). RIP140 siRNA potentiated HOXA5 induction after RA exposure. The magnitude of HOXA5 expression by RIP140 siRNA increased dramatically as compared to control siRNA treated cells. At 56 hours, HOXA5 expression increased ∼20-fold with RA in control siRNA cells and ∼140-fold with RA in RIP140 siRNA cells (Figure 4A). Consistent with data in Figure 3D, HOXA5 expression oscillated in control cells (Figure 4B). The kinetics of HOXA5 expression in RIP140 siRNA cells were altered such that HOXA5 oscillations were replaced by a continual increase in HOXA5 expression over time. This induction had two phases, a modestly increased rate of HOXA5 expression as compared to control cells within in the first 35 hours followed by a steep increase in the expression of HOXA5 thereafter (Figure 4A). These data suggest a potential role for RIP140 in fine-tuning the oscillatory expression of HOXA5 in the continued presence of RA.

### RA target genes differentially respond to RIP140 depletion

Our prior work established that RIP140 siRNA enhances and accelerates retinoid receptor transactivation, expression of RA target genes, and RA-mediated differentiation and growth suppression of pluripotent human embryonal carcinoma cells [11], [12]. Interestingly, microarray studies suggested that distinct subsets of direct RA target genes differentially responded to RIP140 depletion [12]. We wished to validate this apparent selectivity of RIP140.

**Figure 5:**
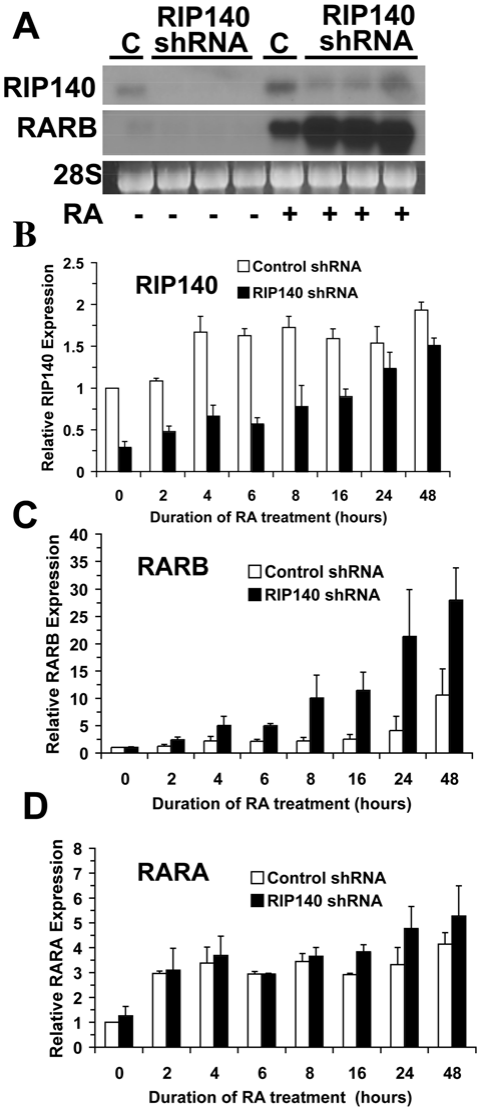
RAR target genes differentially respond to RIP140 depletion. A, Northern analysis depicting the effect of RIP140 shRNA on expression of RIP140 and the known RA target gene RARB. NT2/D1 cells were treated with RIP140 shRNA lentivirus or control shRNA lentivirus and selected in puromycin. Three individual stable pools of RIP140 shRNA treated NT2/D1 cells and one control shRNA treated pool were exposed to RA (1 µM) for 24 hours. The 28S ribosomal RNA served as a loading control. B–D, Quantitative real-time PCR analysis of NT2/D1 shRNA control and NT2/D1 shRNA RIP140 cells exposed to RA (1 µM) for the indicated time points. Data was normalized to GAPDH. Error bars represent the range of the average of duplicate experiments for the expression of B, RIP140; C, RARB; and D, RARA.

NT2/D1 cells were transduced in triplicate with a lentiviral short hairpin RNA (shRNA) targeting RIP140, pLKO.1-RIP140, or a control shRNA targeting GFP, pLKO.1-shGFP. RIP140 shRNA mediated substantial RIP140 depletion under both basal and RA-treated conditions and resulted in a substantial enhancement of RA dependent expression of RARB (Figure 5A). One shRNA pool and one control pool were used to evaluate the kinetics of RARA and RARB induction with RA. Real-time PCR indicated enhancement of RA-dependent RARB expression but no enhancement of RARA expression in RIP140 shRNA cells as compared to control shRNA cells (Figure 5B–5D).

Similarly, RIP140 knockdown using siRNA enhanced RA induction of RARB and HOXA5 but not RARA in NT2/D1 cells (Figure S3 and data not shown). Further, both shRNA and siRNA knockdown of RIP140 enhanced RA induction of RARB but not RARA in the RA-responsive murine lung adenocarcinoma line, ED-1 [22] (data not shown). Thus, in two different cell systems with both siRNA and shRNA knockdown, RIP140 limits RARB and HOXA5 but not RARA expression despite the fact that all three genes are well-characterized, RARE-containing, direct targets of RARs.

### RIP140 distinctly regulates RAR activation as compared to classical NR coregulators

**Figure 6:**
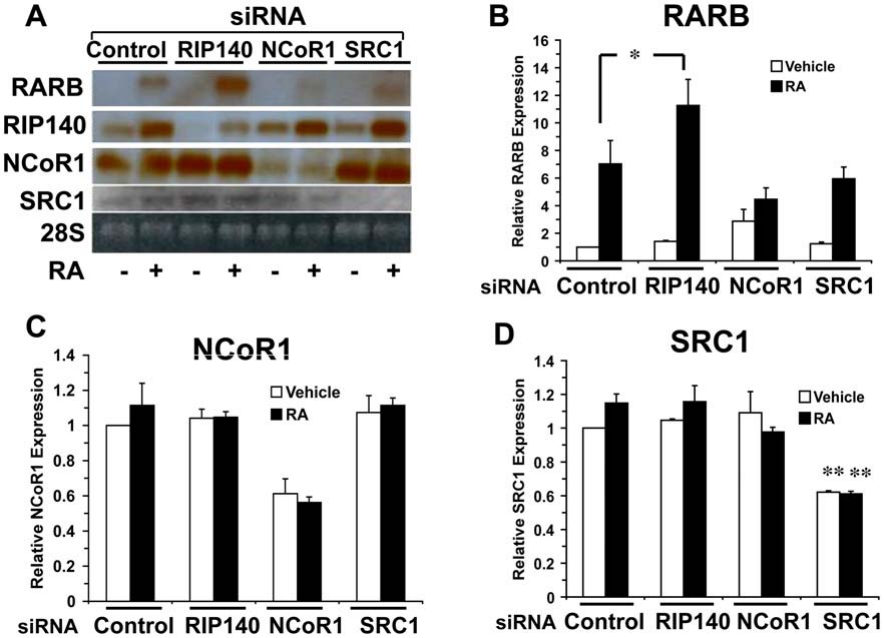
Comparison of the effects of RIP140, NCoR1 and SRC1 depletion on RA-induced RARB expression. A, Northern analysis depicting the effect of control, RIP140, NCoR1 and SRC1 siRNA on expression of the known RA target gene RARB. NT2/D1 cells treated with the indicated siRNA were exposed to RA (1 µM) for 24 hours. The 28S ribosomal RNA served as a loading control. Blot was hybridized to probes specific for RARB, RIP140, NCoR1 and SRC1. Note, only RIP140 siRNA resulted in enhanced RA induction of RARB and only RIP140 is induced with RA. B–D, Quantitative real-time PCR analysis of NT2/D1 cells treated as in (A) and assayed for expression of RARB (B), NCoR1 (C) and SRC1 (D). B and D are the average of triplicate experiments and error bars are S.E.M. *, p<.02 with the paired, two-tailed T test. **, p<.05, with the unpaired, two-tailed T test. (C) is the average of duplicate experiments and error bars depict the range of the two values.

We wished to begin to address whether RIP140 plays a distinct role in the regulation of RA signaling as compared to classic coregulators of nuclear receptors, the p160 family of coactivators (SRC1, SRC2 and SRC3) and the classic corepressors (NCoR1 and NCoR2/SMRT). In contrast to RIP140 siRNA, siRNA to NCoR1 and SRC1 failed to affect RA-dependent RARB induction in NT2/D1 cells (Figure 6A and 6B). In addition, knocking down RIP140, SRC1 or NCoR1 did not greatly influence the expression of one another (Figure 6A, 6C and 6D). While RIP140 is RA inducible and cycles in the constant presence of RA (Figure 2), NCoR1 and SRC1 expression is not responsive to RA and does not oscillate in the constant presence of RA (Figure 6 and Figure S4). These data suggest that RIP140 plays a distinct and non-redundant role in RA-signaling in NT2/D1 cells as compared to SRC1 and NCoR1, which may share roles with the additional known homologs SRC2, SRC3 and NCoR2.

### RIP140 is a general ligand-inducible and ligand-dependent NR corepressor

**Figure 7:**
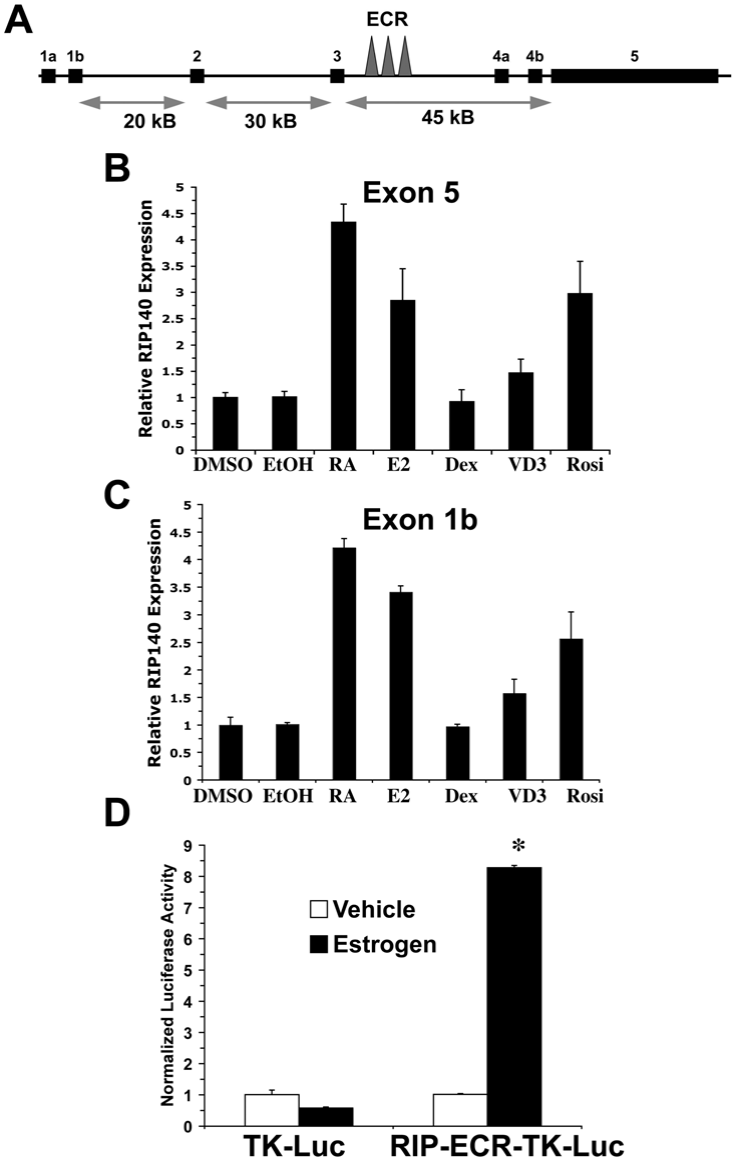
RIP140 expression is induced with RA, estrogen, dexamethasone, and rosiglitazone in MCF7 cells. A, Schematic of the RIP140 gene locus. The protein-coding region of RIP140 is contained within exon 5. A 3.5 kb highly evolutionarily conserved region (ECR) is indicated by the arrowheads. B and C, MCF7 cells were treated with vehicle controls, DMSO or ethanol (EtOH), or RA (1 µM), estrogen (0.1 µM, E2), dexamethasone (1 µM, Dex) or rosiglitazone (1 µM, Rosi) for 24 hours and expression of RIP140 was measured by quantitative real-time PCR with primers within exon 5 (B) or exon 1b (C). Data was normalized to GAPDH. Depicted is that average of triplicate PCR reactions of the same samples and error bars are S.D. D, COS-1 cells were transfected with an ER-alpha expression plasmid and a control thymidine kinase (tk)-liciferase reporter (TK-Luc) or a reporter containing a 550 bp segment of the RIP140 ECR region containing a consensus estrogen receptor response (ERE) (RIP-ECR-TK-Luc). Cells were treated with vehicle or estrogen (0.1 µM) for 24 hours and firefly luciferase activity was determined and normalized to renilla luciferase activity. Bars are the average of triplicate transfections and error bars are S.D. *, p<.0001 with the unpaired two-tailed T test as compared to vehicle treated RIP-ECR-TK-Luc cells. One of two experiments with similar results.

We have shown previously that a four-fold induction of RIP140 by RA occurs within 3 hours of RA treatment and *de novo* protein synthesis is not required for this induction, indicating that RIP140 is a RA target gene [10]. Gene expression profiling has identified the upregulation of RIP140 by RA, estrogens, and vitamin D_3_ [23]–[25]. Estrogens and androgens directly induce RIP140 in MCF-7 breast cancer cells and LNCaP prostate cancer cells, respectively [26], [27]. To investigate the inducibility of RIP140 by nuclear receptor ligands, MCF-7 cells were treated with RA, 17β-estradiol, dexamethasone, vitamin D_3_, rosiglitazone or vehicle control for 24 hours and expression of RIP140 was analyzed by real-time RT-PCR. MCF-7 cells were chosen since they are known to express the cognate receptors for these ligands. RA caused the greatest accumulation of RIP140 mRNA while estradiol and rosiglitazone caused a more modest induction (Figure 7B). A slight induction of RIP140 was also seen with vitamin D_3_ in MCF-7 cells (Figure 7B).

The RIP140 locus spans approximately 100 kb and consists of one coding exon (exon 5) and several upstream non-coding exons (Figure 7A). Multiple RIP140 promoters have been identified and alternative splicing yields multiple RIP140 transcripts [25], [26], [28]. In MCF-7 cells, transcripts containing 1b (Figure 7C) and 1a (data not shown) accumulated after 24 hours of treatment with RA, estrogen or rosiglitazone. Utilizing the Evolutionarily Conserved Regions (ECR) browser [15], we located a 3.5 kb region between exons 3 and 4a that is the most highly evolutionarily conserved region in the RIP140 locus aside from the protein coding sequence contained within exon 5 (Figure 7A). The TRANSFAC algorithm indicated that this region contains sequences matching half or full consensus site response elements for several NRs including ERR, FXR, LXR, ROR, GR and also a consensus ERa/Sp1 type ER responsive element.

We cloned a 550 bp fragment of this region into a thymidine kinase (tk)-luciferase construct and cotransfected this plasmid along with an ERα expression plasmid in COS-1 cells. Estrogen treatment for 24 hours resulted in an approximate 8-fold induction in reporter activity, supporting the presence of a functional ERE in this region (Figure 7D). However, the construct was not responsive to RA or dexamethasone, indicating that functional GREs and RAREs do not exist in this region of the RIP140 locus (data not shown).

**Figure 8:**
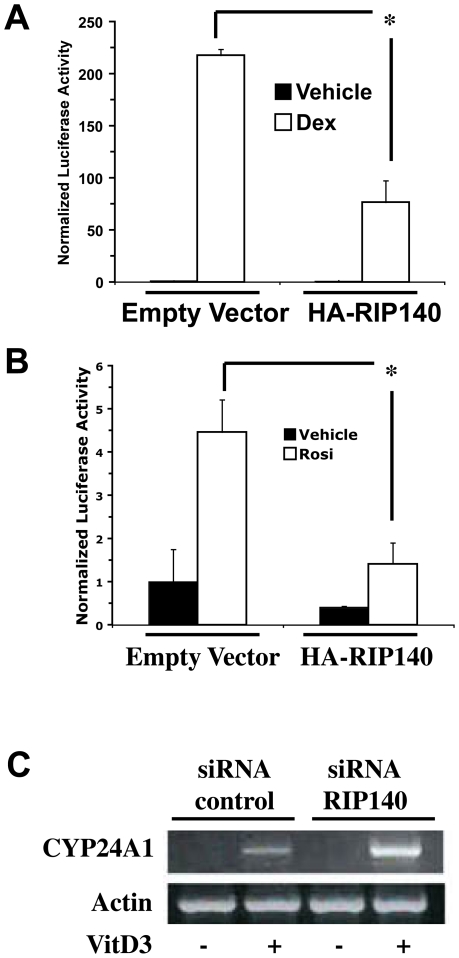
RIP140 represses ligand dependent GR, PPARγ, and VDR activity. A, COS-1 cells were transfected with a GRE-tk-luciferase reporter, a GR expression plasmid and an insertless expression plasmid (empty vector) or RIP140 expressing plasmid (HA-RIP140). Cells were treated with vehicle or dexamethasone (1 µM, Dex) for 24 hours and firefly luciferase activity was measured and normalized to renilla luciferase. Data are the average of triplicate transfections and error bars are S.D. *, p<.005 with the unpaired two-tailed T test. Experiment was repeated with similar results. B, COS-1 cells were transfected with a PPRE-tk-luciferase reporter, PPARγ and RXR expression plasmids and an insertless expression plasmid (empty vector) or RIP140 expressing plasmid (HA-RIP140). Cells were treated with vehicle or rosiglitazone (1 µM, Rosi) for 24 hours and firefly luciferase activity was measured and normalized to renilla luciferase. Data are the average of triplicate transfections and error bars are S.D. *, p<.005 with the unpaired two-tailed T test. One of two experiments with similar results. C, Semi-quantitative PCR analysis of T47D cells transfected with control siRNA or siRNA to RIP140. Cells were treated with Vitamin D_3_ (1 µM) for 24 hours and expression of the VDR target gene, CYP24A1 was determined. Actin expression was used as a control.

There is growing evidence to support a role for RIP140 as a global ligand-dependent NR corepressor [29]. We sought to determine the effect of exogenous RIP140 on the activity of heterologous GR- and PPARγ-responsive promoters (Figure 8). RIP140 cotransfected with GR or PPARγ in COS-1 cells inhibited GRE- and PPRE-dependent activation by dexamethasone and rosiglitazone, respectively (Figure 8A and 8B). In addition, induction of CYP24A1, an endogenous vitamin D_3_ target gene with a well-defined VDRE, was enhanced with RIP140 siRNA in T47D cells (Figure 8C). These results, along with the data on RIP140 inducibility by NRs (Figure 7) supports prior studies suggesting that RIP140 may be a general ligand-dependent and ligand-inducible repressor of NRs [6], [23]–[31]. These properties suggest that RIP140 has the potential to be involved in oscillatory transactivation of other NRs in addition to RARs.

## Discussion

Oscillations are important homeostatic devices [32]. Negative feedback drives many oscillatory events and can lend both stability and plasticity to a biological system [32], [33]. Oscillations in NR activity may allow for control of hormone responses through continual monitoring of ligand availability and enable NR signaling to be poised to appropriately adjust to rapid fluctuations. The temporal complexity of differentiation suggests that elucidation of the timing of gene expression in tumor cells is important to understand the basis of the therapeutic effects of RA. The periodic nature of RA signaling may be an important consideration for the more efficacious use of retinoids, for example in conjunction with targeting coregulators like RIP140.

The past decade has revealed that NR activation is highly dynamic. Periodic activation of NR binding, coregulator recruitment, preinitiation complex assembly, and gene transcription in the constant presence of ligand has been described for ER, GR, and VDR [5], [17]–[19], [34]–[38]. These transcription cycles occur with a short periodicity of 20–60 minutes and are mediated by acetylation, phophorylation, proteasomal degradation and chromatin remodeling [5], [17]–[19], [34]–[38]. We provide evidence for an additional, extended oscillation of retinoid receptor activation and RA target gene expression in the constant presence of RA that is long timed (24–35 hours) suggestive of a transcriptional-translational loop. Oscillatory retinoid receptor activity was evident in both proliferating and nonproliferating cells, in human and murine cell lines, with RA and RAR/RXR selective agonists, and in cell models dependent on endogenous as well as episomal and stably integrated promoters. In addition, the long paced cycles were partially perturbed by overexpression and depletion of the ligand-dependent corepressor, RIP140. Since RIP140 is acutely inducible by RA and represses ligand bound RAR activity we speculate that it is poised to potentially pace or fine-tune the frequency or amplitude of these cycles.

Of note, endogenous levels of HOXA5 and to a lesser extent RARB, tended to increase with each oscillation in the continued presence of RA in the absence and presence of RIP140 siRNA (Figure 3 and Figure 4). While accumulation of mRNA may be expected to occur with each cycle, it is not clear why hnRNA would increase with each cycle unless the rate of transcription was also increasing with each oscillation. Further studies are necessary to understand these findings.

It has been shown previously that short-paced oscillations of ER, GR, and AR activity correlate with cyclic recruitment of coregulators, (including RIP140) to the promoters of NR target genes [5], [17]–[19], [34]–[38]. However, the temporal nature of coregulator recruitment to RARs and other class II receptors that are resident at promoters in the absence of ligand has been less well characterized. It is currently unclear whether the long paced oscillations we have observed are specific for RARs or coexist with short paced cycles that have been shown for ER, GR and AR. Although not addressed in the current study, it is conceivable that each short-term cycle of coregulator recruitment (20–60 min) affords the opportunity to monitor the relative ratios of coregulators changing over a macro time scale subject to long-term changes in environmental conditions. Thus it will be important in the future to carefully monitor the recruitment of coregulators in our system using techniques such as chromatin immunoprecipitation. Interestingly, Wang *et al*. showed that in contrast to ER complexes which form within minutes and recycle hourly, complexes at AR promoters slowly form over 16 hours and then slowly decline [39].

Although RA is required for proper maintenance of epithelial tissues, the binary nature of RA-induced differentiation, in which cells commit to differentiate upon a defined time interval, may not be the ideal setting for the characterization of long-paced oscillations of NR activity. However, the dynamic patterns we have uncovered may be particularly relevant to other NRs that receive constant signals such as orphan NRs (for example, ERRs), steroid receptors that receive fairly constant hormonal stimulus over days (ER, AR and PR), and NRs that receive signals from the diet or intracellular metabolism (PPARs, FXRs, LXRs). It is noteworthy the we and others have shown that RIP140 is induced by a number of NRs including RAR, ER, PPARγ, AR and ERR and that RIP140 can in turn repress these and other NRs (Figure 7)[6], [23]–[31], [40]. Particularly intriguing are the potential two-way interactions between RIP140 and ERR-alpha and PPARγ, since these receptors along with RIP140 are known to be key regulators of cellular metabolism *in vivo* [6], [28], [40]–[45]. It will be of interest to discover in the future if other long-paced NR oscillations exist and whether they integrate with rhythmic biological processes known to involve NRs, for example, the menstrual cycle, the circadian clock and digestive and metabolic cycles.

Although we have provided evidence that RIP140 may influence long-paced oscillations in NR activity, it is unclear whether RIP140 is required for these oscillations or rather is involved in fine-tuning their timing, frequency and amplitude. Further, the precise timing of the oscillations differs depending on the cell system (integrated versus episomal reporter) and assay (real time PCR versus reporter) which may reflect differences in the precision inherent with each technique. A more thorough understanding of the dynamics of RIP140 expression and processing would further support this model. RIP140 is known to be extensively modified posttranslationally [46].

We have previously identified selectivity of RIP140 in the regulation of RA target genes by employing microarrays on a genome-wide scale at a 24-hour time point [12]. Consistent with our microarray results, RARB and HOXA5 induction was robustly potentiated by RIP140 depletion while RARA induction was unaffected. Similar results were obtained in two different cancer cell lines using both transient siRNA and stable shRNA. The molecular basis of this selectivity remains unknown. The well-characterized RAREs in the RARA and RARB promoters are highly similar, both consist of tandem direct repeat half sites separated by 5 bp (DR5) and only one base differs across the entire 12 bp consensus [47], [48]. It is reasonable to speculate that chromatin structure at different promoters may underlie RIP140 binding selectivity. RIP140 has been shown to be recruited to the RARB promoter [49], [50], but no studies have yet to investigate whether RIP140 is recruited to the RARA promoter. Alternatively, RIP140 may differentially affect retinoid receptor subtypes. Selective gene regulation by RIP140 suggests that RA target genes may undergo distinct patterns of dynamic gene expression relevant to the growth suppressive and differentiating effects of RA.

In summary, we have shown that RAR activity and RIP140 and RA target gene expression undergo long-paced oscillations in the constant presence of RA. Further we provide evidence to allow us to speculate a potential modulatory role for RIP140 in pacing and fine-tuning these low-frequency cycles of NR activity. Tumor cell differentiation is a highly dynamic process, and as RIP140 limits the effectiveness of RA-mediated differentiation, further characterization of the role of RIP140 in RA signaling dynamics will afford a more accurate understanding of the beneficial therapeutic actions of retinoids. The regulation of RIP140 by diverse hormonal signals and its general corepression of NRs suggest that RIP140 oscillations may be relevant to the temporal regulation of other hormone signaling events in distinct physiologic and pharmacologic contexts.

## Supporting Information

Figure S1 Primers for PCR. Sequences are 5′-3′. H, human; m, mouse; hn, heteronuclear PCR. All primers were used for quantitative PCR with SYBR Green reagent with the exception of those with (*) which were used for semiquantitative PCR.
(2.09 MB TIF)

Figure S2 Stably integrated RAREs support oscillations in RAR activity in the constant presence of RA or RAR and RXR agonists. A, U2OS cell pools containing random, stably integrated RARE-tk-luciferase were grown to confluence and serum starved for 2 days prior to the addition of media containing RA (10 µΜ) or the RXR pan agonist bexarotene (5 µM) + the RAR pan agonist TTNPB (5 µM) (Bex + TTNPB). An additional group of cells were pretreated with alpha-amanitin (2.5 µM) for 2 hours prior to the addition of bexarotene and TTNPB (Am + Bex + TTNPB). Upon addition of retinoids cells were placed in a LumiCycle luminometer and luciferase activity was measured every 10 minutes for 80 hours. Each data line is the average of triplicate plates and data was baseline subtracted using LumiCycle software. Inset, data in (A) with zoomed scaling. B, NIH-3T3 cell pools containing random, stably integrated RARE-tk-luciferase were grown to confluence and serum starved for 2 days prior to the addition of bexarotene (5 µM) and TTNPB (5 µM). Cells were transferred to a LumiCycle luminometer and luciferase activity was measured every 10 minutes for 95 hours. Each data line represents one of four separate replicates and data was baseline subtracted. Inset, data in (B) with zoomed scaling. The experiment was performed twice with similar results.
(4.27 MB EPS)

Figure S3 RAR target genes differentially respond to RIP140 siRNA. NT2/D1 cells were treated with RIP140 siRNA or control siRNA and treated with RA (1 µµM) for the indicated time points. Quantitative real-time PCR analysis was performed for A, RARA and B, HOXA5. Data was normalized to GAPDH. Error bars represent the range of the average of duplicate experiments.
(2.30 MB TIF)

Figure S4 NCoR1 and SRC1 do not oscillate in the continued presence of RA. NT2/D1 cells were pretreated with alpha-amanitin (2.5 µM) for 2 hours and then treated with RA (1 µM for the indicated time points. NCoR1 (A) and SRC1 (B) expression was monitored by quantitative real-time PCR. One of two experiments with similar results is presented. Data was normalized to GAPDH. Trend line is the 5th order polynomial.
(2.51 MB TIF)
