# Supplementary figures and images for: Retinoic Acid Mediates Long-Paced Oscillations in Retinoid Receptor Activity: Evidence for a Potential Role for RIP140
Source: PLoS One. 2009 Oct 28;4(10):e7639. doi: 10.1371/journal.pone.0007639 (PMC2763268; doi:10.1371/journal.pone.0007639)

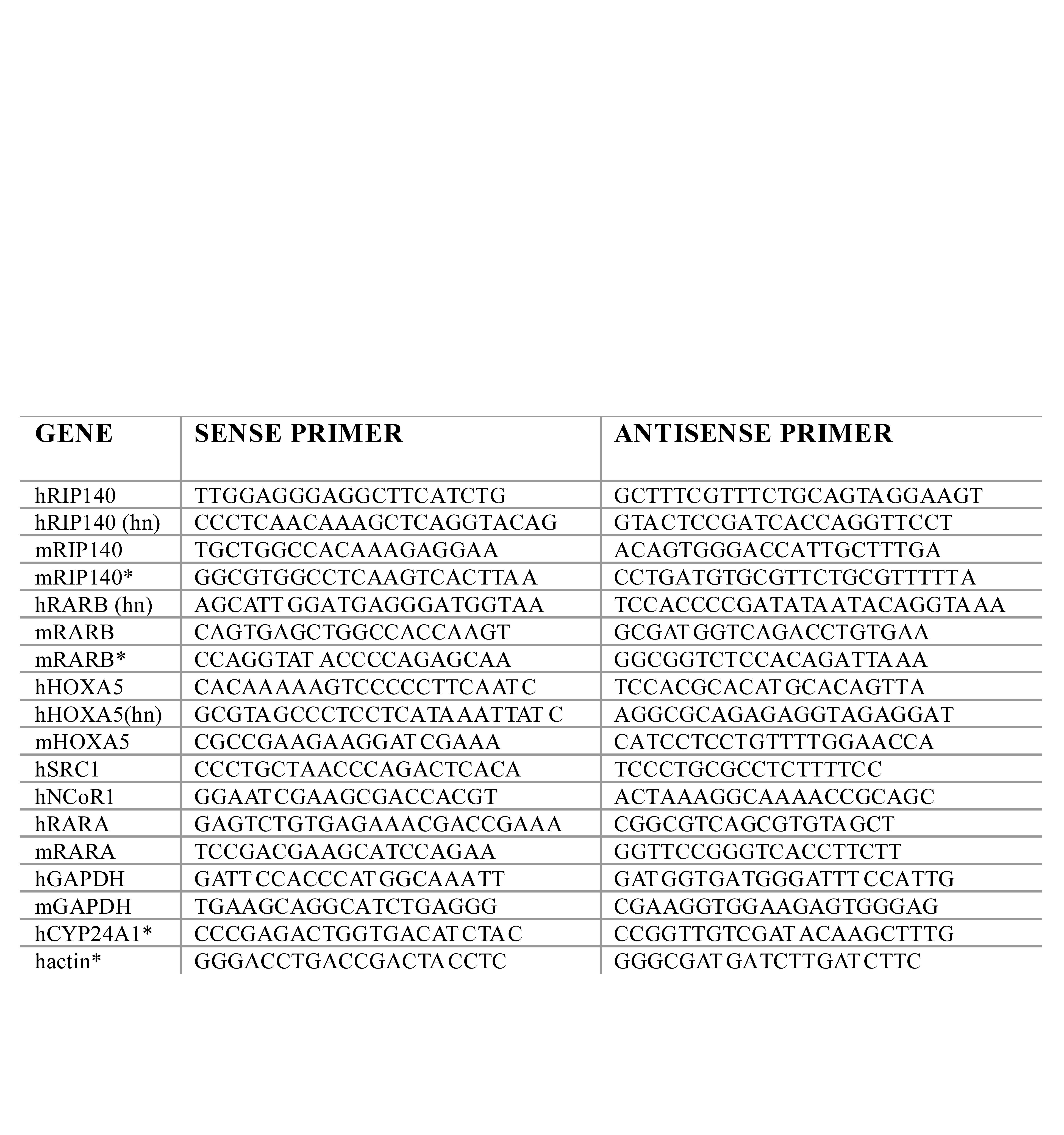

Supplement: Figure S1 — Primers for PCR. Sequences are 5′-3′. H, human; m, mouse; hn, heteronuclear PCR. All primers were used for quantitative PCR with SYBR Green reagent with the exception of those with (*) which were used for semiquantitative PCR. (2.09 MB TIF) [file pone.0007639.s001.tif]

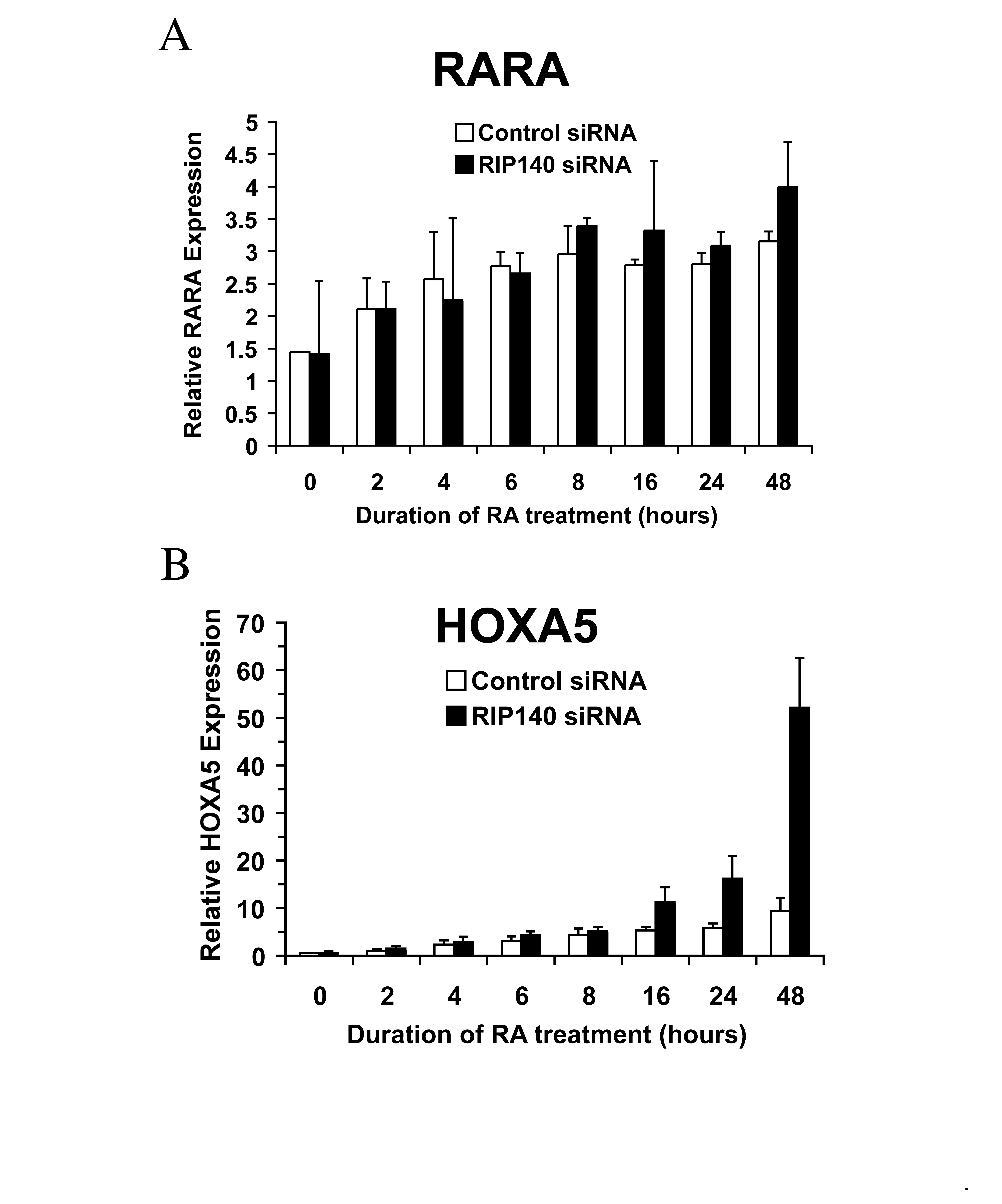

Supplement: Figure S3 — RAR target genes differentially respond to RIP140 siRNA. NT2/D1 cells were treated with RIP140 siRNA or control siRNA and treated with RA (1 µµM) for the indicated time points. Quantitative real-time PCR analysis was performed for A, RARA and B, HOXA5. Data was normalized to GAPDH. Error bars represent the range of the average of duplicate experiments. (2.30 MB TIF) [file pone.0007639.s003.tif]

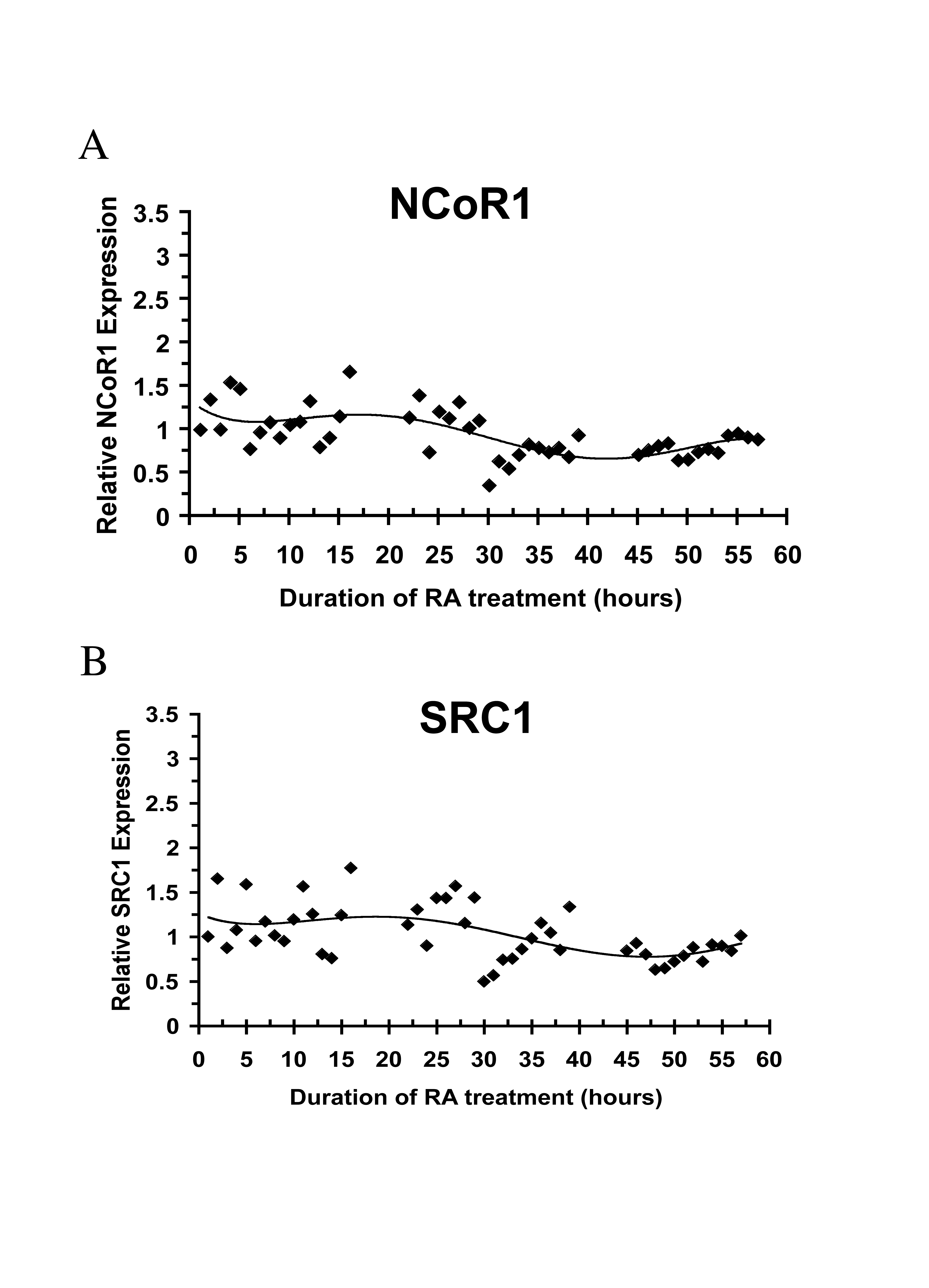

Supplement: Figure S4 — NCoR1 and SRC1 do not oscillate in the continued presence of RA. NT2/D1 cells were pretreated with alpha-amanitin (2.5 µM) for 2 hours and then treated with RA (1 µM for the indicated time points. NCoR1 (A) and SRC1 (B) expression was monitored by quantitative real-time PCR. One of two experiments with similar results is presented. Data was normalized to GAPDH. Trend line is the 5th order polynomial. (2.51 MB TIF) [file pone.0007639.s004.tif]
